# Disruption of quorum sensing and biofilm formation by lawsone in gram-negative bacteria

**DOI:** 10.1128/spectrum.01187-26

**Published:** 2026-07-16

**Authors:** Razique Anwer

**Affiliations:** 1 Department of Pathology, College of Medicine, Imam Mohammad Ibn Saud Islamic University (IMSIU) 248361, Riyadh, Saudi Arabia; Universita degli Studi Roma Tre Dipartimento di Scienze, Rome, Italy

**Keywords:** antimicrobial resistance, lawsone, quorum-sensing inhibition, antivirulence strategy, biofilm, molecular simulation

## Abstract

Antimicrobial resistance is a major global health threat, exacerbated by bacterial virulence mechanisms such as quorum sensing (QS) and biofilm formation, which enhance pathogenicity and reduce antibiotic susceptibility. Targeting QS offers a promising antivirulence strategy that may attenuate pathogenic traits without imposing selective pressure. In this study, lawsone was evaluated for anti-QS and antibiofilm activity against *Chromobacterium violaceum*, *Pseudomonas aeruginosa*, and *Serratia marcescens*. Lawsone significantly inhibited QS-regulated virulence factors, with reductions in violacein production *in C. violaceum* (90.62%) at 250 µg/mL (1.43 mM). There was also a reduction in pyocyanin and pyoverdin in *P. aeruginosa* (87.90% and 81.97%, respectively) and prodigiosin in *S. marcescens* (~83%) at 500 µg/mL (2.87 mM) lawsone. Additionally, lawsone suppressed protease and elastase activities, as well as rhamnolipid production in *P. aeruginosa*. Biofilm formation was inhibited in a dose-dependent manner, reaching 84.28% in *C. violaceum*, 76.22% in *P. aeruginosa*, and 56.27% in *S. marcescens* at respective sub-MICs. Computational analyses supported these findings; docking showed stable binding of lawsone to QS regulators, including CviR (−7.8 kcal/mol), LasR (−8.5 kcal/mol), and SmaR (−7.7 kcal/mol). Molecular dynamics simulations confirmed the stability of these complexes, with root mean square deviation values of 0.200 ± 0.016 nm (CviR-lawsone), 0.238 ± 0.036 nm (LasR-lawsone), and 0.632 ± 0.061 nm (SmaR-lawsone). Molecular Mechanics Poisson–Boltzmann Surface Area (MM-PBSA) calculations further demonstrated favorable binding. To my knowledge, this is the first study to systematically demonstrate the anti-QS and antibiofilm potential of lawsone across multiple clinically relevant gram-negative pathogens using an integrated experimental and computational approach. This work provides possible mechanistic insights into lawsone-QS regulator interactions, highlighting its potential as a broad-spectrum antivirulence agent targeting conserved QS systems.

**IMPORTANCE:**

Antimicrobial resistance is a growing global threat, driven in part by bacterial virulence mechanisms such as quorum sensing (QS) and biofilm formation. Targeting QS offers an alternative strategy to reduce pathogenicity without promoting resistance. This study shows that lawsone effectively inhibits QS-regulated virulence factors and biofilm formation in gram-negative pathogens, like *Pseudomonas aeruginosa*, *Serratia marcescens*, and *Chromobacterium violaceum*. These findings highlight lawsone as a promising antivirulence agent that could complement existing therapies for managing biofilm-associated and drug-resistant infections.

## INTRODUCTION

Antimicrobial resistance (AMR) is a growing health problem. Bacteria that no longer respond to common antibiotics cause longer illnesses, more deaths, and higher treatment costs. New drugs are hard to develop, and drug resistance often appears soon after use (1). Because of this, researchers look for alternative ways to control infections that do not solely rely on killing bacteria (2). One promising idea is to block bacterial communication called quorum sensing (QS). QS lets bacteria sense how many of them are nearby and switch on group genes/behaviors when a threshold is reached. Many harmful traits like toxin release, enzyme secretion, movement on surfaces, and biofilm formation are controlled by QS (3). If we can stop QS, bacteria become less able to cause disease even if they are still alive. This antivirulence approach may reduce selection pressure for resistance as it does not directly kill cells. Biofilms are a major reason why infections become chronic and difficult to treat. In a biofilm, bacteria live inside a sticky matrix of polysaccharides, proteins, and DNA (4). This matrix slows antibiotic penetration and protects cells from the immune system. Biofilm cells can tolerate antibiotics many times more than free-living cells (5). Therefore, preventing biofilm formation or disrupting established biofilms is an important strategy to improve infection control.

Natural small molecules are becoming an emerging source of QS inhibitors. Many plant compounds can interfere with QS signaling or block the receptor proteins that detect autoinducers (6). These compounds often act at sub-inhibitory concentrations (sub-MICs) and reduce virulence without strong bactericidal effects. Bioinformatic tools like molecular dynamics (MD) simulations and molecular docking help to predict how small molecules bind to QS regulators and to understand the stability of these interactions. Lawsone (2-hydroxy-1,4-naphthoquinone) is a natural naphthoquinone found in henna and some medicinal plants (7). It has known antioxidant, anti-inflammatory, and antimicrobial properties. However, its effect on QS and biofilm formation in gram-negative pathogens has not been fully explored. Because lawsone is small, aromatic, and moderately hydrophobic, it is a good candidate to test for binding to LuxR-type QS receptors and for antivirulence activity.

In this study, the anti-QS and antibiofilm effects of lawsone were tested against *Chromobacterium violaceum*, *Pseudomonas aeruginosa*, and *Serratia marcescens*. The QS-regulated virulence factors, extracellular enzymes, motility, and biofilm formation were tested at sub-MICs of lawsone. The molecular docking, MD simulations, and MM-PBSA were performed to examine how lawsone interacts with the LuxR-type regulators CviR, LasR, and SmaR. By targeting QS and virulence rather than bacterial survival, lawsone offers an alternative strategy to conventional antibiotics. Further work with clinical isolates and *in vivo* models is needed to assess safety, pharmacokinetics, and therapeutic potential. This study provides a foundation for developing lawsone or related naphthoquinones as antivirulence agents to help manage biofilm-associated and chronic infections.

## MATERIALS AND METHODS

### Bacterial strains and chemicals

*Serratia marcescens* ATCC 13880, *Pseudomonas aeruginosa* PAO1, *Chromobacterium violaceum* 12472, and *C. violaceum* CV026 were used in this study. Unless otherwise stated, the cultures were grown in Luria-Bertani (LB) broth, and experiments were performed in this medium. Lawsone (H46805) was obtained from Sigma-Aldrich, USA, with 97% purity, and was used directly without any further processing. Trichloroacetic acid (TCA), orcinol, Elastin-Congo Red (ECR), azocasein, and 2,3,5-triphenyl-tetrazolium chloride (TTC) solution were procured from Sigma-Aldrich, USA. The organic solvents required for the experiments were sourced from Thermo Fisher Scientific. All other chemicals and reagents used in this work were of analytical grade and used directly without any additional purification.

### MIC determination and growth curve analysis

The MIC of lawsone against bacterial strains was determined using TTC dye with twofold serial dilutions ranging from 15.625 to 2,000 µg/mL in 96-well microtiter plates, following standard procedure (8). The growth curve was assessed in 50 mL LB medium in 100 mL flasks. Briefly, 0.2 mL bacterial inoculum from a 6-h-grown culture of *S. marcescens*, *P. aeruginosa*, and *C. violaceum* was added to the flasks. For the treatment group, *C. violaceum* was grown in the presence of 250 µg/mL lawsone, whereas *S. marcescens* and *P. aeruginosa* were treated with 500 µg/mL lawsone. The cultures were incubated at their respective optimum growth temperatures. Aliquots of 1 mL were collected at 3-h intervals, and the optical density was recorded at 600 nm.

The viable cell counts were measured to assess the effect of lawsone on bacterial viability. The culture growth and treatment with lawsone (250 µg/mL for *C. violaceum* and 500 µg/mL for *S. marcescens* and *P. aeruginosa*) were performed as mentioned in the above section. After 24 h of incubation, the cultures were serially diluted in sterile PBS and plated onto LB agar plates. The plates were incubated overnight, and the number of colonies was counted. The number of colonies appearing on the plates was converted to log_10_CFU/mL.

### Violacein estimation in *C. violaceum*

The extraction of violacein pigment was performed in dimethyl sulfoxide (DMSO) (9). *C. violaceum* was cultured overnight in LB medium, both in the absence and presence of lawsone at sub-MIC levels (25–250 µg/mL). After incubation, 1 mL of the culture was centrifuged to separate the insoluble violacein pigment. The pellet obtained was resuspended in 1 mL of DMSO and mixed thoroughly by vortexing until the pigment dissolved properly. The sample was again centrifuged at 10,000 rpm to remove any remaining bacterial cells. The optical density of the supernatant containing violacein was measured at 585 nm.

The effect of lawsone on violacein production in *C. violaceum* CV026 was tested using a microplate assay. In brief, bacterial inoculum, LB medium, lawsone (25–250 µg/mL), and *N*-hexanoyl-L-homoserine lactone (C_6_-HSL) were added to make a final volume of 200 µL in 96-well plates. The plates were incubated at 27°C for 24 h. After incubation, cultures were transferred into 0.5 mL tubes and centrifuged to collect the cells. The violacein pigment was extracted by resuspending the pellet in 200 µL of DMSO and vortexing thoroughly. The mixture was centrifuged again to remove cell debris, and the absorbance of the supernatant was measured at 585 nm to quantify pigment production. The percent inhibition was calculated with respect to the C_6_-HSL-supplemented *C. violaceum* CV026 group.

### Examination of *P. aeruginosa* PAO1 virulence factors

Pyocyanin production was tested in *Pseudomonas* broth containing potassium sulfate (10 mg/mL), magnesium chloride (1.4 mg/mL), and peptone (20 g/L) (10). Cultures of *P. aeruginosa* were incubated for 18 h without and with sub-MICs of lawsone (50–500 µg/mL). After centrifugation, 5 mL of culture supernatant was extracted in 3 mL of chloroform, and the organic phase was collected and mixed with 1 mL of 0.2 N HCl. The mixing of these two phases turned the 0.2 N HCl into pink/red. The aqueous phase was separated, and its absorbance was measured at 520 nm. Pyocyanin levels were obtained using the factor 17.072.

Pyoverdin estimation followed the reported method (11). Overnight cultures grown with and without lawsone were centrifuged, and 0.1 mL of supernatant was mixed with 0.9 mL of Tris-HCl buffer (pH 7.4). Fluorescence was recorded at an excitation of 400 nm and an emission of 460 nm.

Protease activity was determined using the azocasein degradation method (12). Cell-free supernatants from untreated and lawsone-treated cultures were collected. A 0.1 mL aliquot of supernatant was mixed with 1 mL of 0.3% azocasein prepared in 50 mM Tris-HCl buffer (pH 7.5) containing 0.5 mM CaCl_2_. The reaction mixture was incubated at 37°C for 15 min. The reaction was stopped by adding 0.5 mL TCA (10%). Samples were centrifuged to remove precipitated proteins. The absorbance of the clear supernatant was measured at 400 nm.

Elastinolytic activity was determined using ECR as substrate (13). A 0.1 mL culture supernatant was added to 0.9 mL ECR buffer (1 mM CaCl_2_ and 5 g/L ECR in 100 mM Tris). The sample was placed in an incubator for 3 h at 37°C with shaking, followed by the addition of 1 mL phosphate buffer (0.1 mM, pH 6.0). Samples were cooled on ice for 30 min, centrifuged, and the absorbance of the supernatant was measured at 495 nm.

Rhamnolipid quantification was carried out using the orcinol assay. Culture supernatant (0.3 mL) was mixed with diethyl ether (0.6 mL). The diethyl ether was collected, and it was evaporated at 37°C. The dried residue was dissolved in 100 µL deionized water. To this sample, 0.9 mL orcinol (0.19% in 53% H_2_SO_2_) was added. The reaction mixture was incubated for half an hour at 80°C, cooled at room temperature for 15 min, and the optical density was measured at 421 nm.

The qRT-PCR was performed to evaluate the effect of lawsone on transcription of QS genes in *P. aeruginosa* PAO1. Overnight cultures of *P. aeruginosa* were grown to an OD_600_ of 0.8, diluted 1:10 into fresh LB broth (10 mL), and incubated either in the absence or presence of 500 µg/mL lawsone for overnight growth. Cells were harvested, and total RNA was isolated using TRIzol reagent (Invitrogen). Extracted RNA was dissolved in diethyl pyrocarbonate-treated water. The contamination of genomic DNA was removed by DNase I treatment. The cDNA was synthesized using QuantiTect Reverse Transcription Kit (Qiagen) following the manufacturer’s instructions, and it was stored at −20°C. In this study, four QS genes, *lasI*, *lasR*, *rhlI*, and *rhlR,* were examined, and their details were obtained from a previously published article (14). The qPCR cycling conditions were as follows: initial denaturation at 95°C for 10 min, then 40 cycles of 95°C for 15 s and 60°C for 1 min. Relative mRNA levels in treated and control samples were calculated using the comparative cycle threshold method, with 16S rRNA serving as the housekeeping gene for normalization.

### Examination of *S. marcescens* virulence factors

Prodigiosin was extracted from *S. marcescens* cultures grown without and with lawsone (50–500 µg/mL) (15). Cells were harvested by centrifugation, and the pellet was treated with 1 mL of acidified ethanol (4 mL of HCl [1 M] + 96 mL of ethanol). Following extraction, samples were centrifuged to remove debris. Optical density of the supernatant containing pigment (prodigiosin) was measured at 534 nm.

Protease activity was tested using the azocasein degradation assay (12). Cell-free supernatants (0.1 mL) were mixed with 1 mL azocasein (0.3%). The sample was then incubated for 15 min at 37°C. The reaction was stopped by adding 0.5 mL of 10% TCA, followed by centrifugation at 12,000 rpm for 10 min. The absorbance of the supernatant was recorded at 400 nm.

Swarming motility in *S. marcescens* was assessed on LB agar plates containing 0.5% agar. A 5 µL spot of overnight culture was placed on the plate surface and air-dried. Plates were prepared with different sub-MICs of lawsone, while untreated plates served as a control. After incubation at 37°C for 18 h, the motility was examined by recording the migration zone diameter. The percent inhibition is represented relative to the untreated control.

### Biofilm inhibition assay

Biofilm formation was evaluated in 96-well microtiter plates using crystal violet staining. Overnight cultures were inoculated into wells containing 150 µL medium without or with respective sub-MICs of lawsone. Plates were incubated for 24 h. After incubation, the medium was removed to eliminate planktonic cells, and wells were rinsed three times with sterile buffer. Wells were air-dried for 20 min, followed by staining with 200 µL crystal violet for 20 min. Excess dye was washed off with buffer, and the bound stain was solubilized in 90% ethanol. Absorbance was measured at 620 nm using a microplate reader.

For microscopic analysis of biofilm inhibition, overnight cultures of test bacteria were inoculated into 12-well plates containing LB medium and sterile glass coverslips, without or with respective highest sub-MICs of lawsone (250 µg/mL for *C. violaceum* and 500 µg/mL for *P. aeruginosa* and *S. marcescens*). After 24 h of incubation, coverslips were rinsed with sterile phosphate buffer to remove loosely attached cells, stained with 0.1% crystal violet, and air-dried. Biofilms were visualized under a light microscope equipped with a color VGA camera. Images were captured at 40× magnification.

### Molecular docking

Molecular docking was performed to study how lawsone interacts with QS proteins. Three target proteins, namely *C. violaceum*’s CviR, *P. aeruginosa’*s LasR, and *S. marcescens*’s SmaR, were selected. The lawsone structure (CID: 6755) was taken from PubChem. Ligand preparation was carried out with MGL Tools 1.5.6, allowing flexibility, and saved as a PDBQT file. The docking was done using AutoDock Vina, which provides higher speed and accuracy compared to AutoDock4 (16, 17). Protein structures were obtained from the RCSB Protein Data Bank. The structure of SmaR was modeled using MODELLER 10.4. Prior to docking, proteins were processed by removing water, adding non-polar hydrogen atoms, and placing Kollman charges with the help of MGL Tools 1.5.6 (18). Finally, the proteins were saved as PDBQT. Interaction analysis of docked complexes was carried out using PyMOL and Discovery Studio.

### Molecular dynamics and simulation

MD simulations were carried out using GROMACS 2018.1 with amber99sb-ILDN force field (19, 20). Protein topologies were generated in GROMACS, while ligand topology for lawsone was prepared with the Antechamber module of AmberTools24 using the AM1-BCC method for charge calculation (21). Protein and ligand topologies were combined, and each complex was placed in a cuboidal box filled with TIP3P water. Counter ions were added to neutralize the system, and NaCl was introduced to achieve a 150 mM physiological salt concentration. A minimum distance of 1.0 nm between the complex and box edges was kept, maintaining the periodic boundary conditions (PBCs). Energy minimization was performed using the steepest descent algorithm. The equilibration was performed in two steps. First, NVT ensemble at 310 K for 500 ps using the V-rescale thermostat (22), followed by NPT ensemble at 1 bar and 310 K for 0.5 ns using Parrinello–Rahman barostat (23). Production runs were performed for 100 ns, saving 5,000 trajectory frames. Each simulation was repeated in triplicate. PBC corrections were applied before analysis. Binding free energies of protein-lawsone complexes were calculated using the MM-PBSA method (24).

### Statistical analysis

All *in vitro* virulence factor experiments were performed using three independent biological replicates, while biofilm assays were performed using four independent biological replicates. All *in vitro* experiments were also performed with two technical replicates, and the data were averaged to obtain each biological replicate. Results are expressed as mean ± standard deviation. Statistical significance was determined using Student’s *t*-test. *P-value < 0.05; **P-value < 0.01; *** *P-*value < 0.001; and ns, statistically insignificant (*P-*value *>* 0.05). MD simulation data are presented as the mean of three independent runs with standard deviations.

## RESULTS AND DISCUSSION

### MIC and the effect of sub-MIC of lawsone on bacterial growth

**Fig 1:**
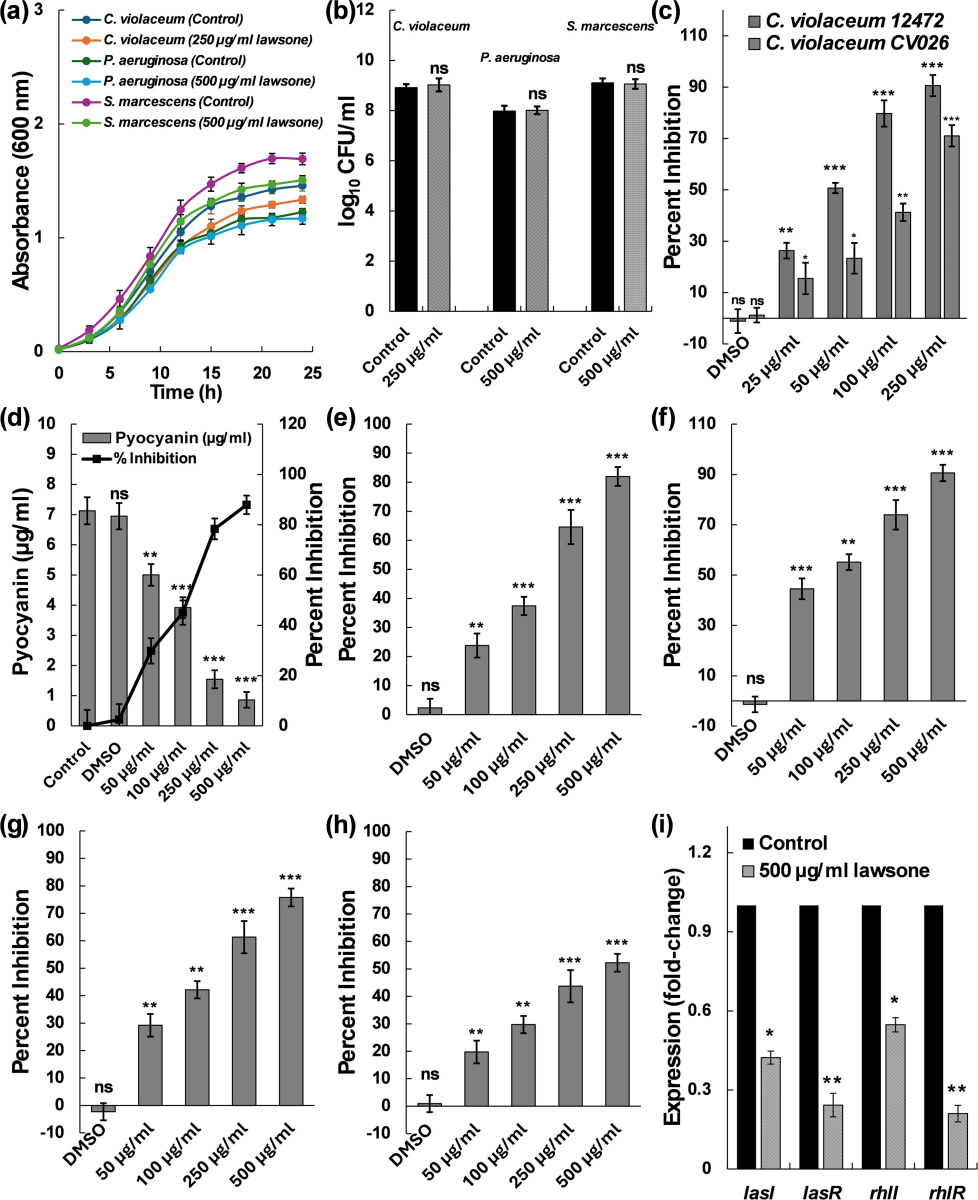
(**a**) Effect of lawsone on the growth kinetics of *C. violaceum* 12472, *P. aeruginosa* PAO1, and *S. marcescens* ATCC 13880. (**b**) Effect of lawsone on the cell viability of *C. violaceum*, *P. aeruginosa*, and *S. marcescens*. (**c**) Inhibition of violacein in *C. violaceum* 12472 and *C. violaceum* CV026 by lawsone. (**d**) Inhibition of pyocyanin in *P. aeruginosa* by lawsone. (**e**) Inhibition of pyoverdin in *P. aeruginosa* by lawsone. (**f**) Inhibition of protease in *P. aeruginosa* by lawsone. (**g**) Inhibition of elastase in *P. aeruginosa* by lawsone. (**h**) Inhibition of rhamnolipid in *P. aeruginosa* by lawsone. (**i**) Effect of lawsone on the transcription of quorum-sensing-related genes in *P. aeruginosa*. Results are expressed as mean ± SD. Statistical significance was determined using Student’s *t*-test. *P-value < 0.05; **P-value < 0.01; ***P-value < 0.001; and ns, statistically insignificant (*P*-value > 0.05). 25 µg/mL, 0.143 mM; 50 µg/mL, 0.287 mM; 100 µg/mL, 0.574 mM; 250 µg/mL, 1.435 mM; and 500 µg/mL, 2.87 mM.

The MIC of lawsone was determined using the TTC-based microdilution method. The MIC of lawsone was observed as 2 mg/mL for *C. violaceum* 12472, while no growth inhibition was observed in *S. marcescens* ATCC 13880 or *P. aeruginosa* PAO1 within the tested concentration range. The effect of sub-MIC of lawsone was tested on the bacterial growth kinetics as shown in Fig. 1a. Lawsone at sub-MIC (500 µg/mL) did not affect the growth of *P. aeruginosa*, with only a negligible change in OD_600_ after 24 h. However, reductions of 8.43% and 11.01% in OD_600_ were observed in *C. violaceum* treated with 250 µg/mL lawsone and *S. marcescens* treated with 500 µg/mL lawsone, respectively. It was worth noting that the pigment production in treated cultures of *C. violaceum* 12472 and *S. marcescens* ATCC 13880 was remarkably reduced. The presence of dark pigments can hamper the absorbance of light in cultures of these pigment-producing bacteria. Therefore, it was explored whether the reduction in OD_600_ was due to growth inhibition or pigment inhibition. For this, the CFU was determined both in control and lawsone-treated conditions. Insignificant differences were found in log_10_CFU/mL of all three tested bacteria at the above-mentioned sub-MICs (Fig. 1b). The data confirmed that the tested sub-MICs do not affect bacterial viability. All subsequent experiments of QS and biofilm development were carried out at concentrations below MIC (abbreviated as sub-MIC).

### Lawsone inhibits violacein pigment in *C. violaceum*

In *C. violaceum* 12472, violacein biosynthesis is regulated by QS, where CviI synthesizes acyl homoserine lactones (AHLs) that are detected by CviR. Once a threshold concentration of AHLs is reached, this interaction activates transcription of violacein biosynthetic genes (25). A clear dose-dependent reduction in violacein pigment was observed with increasing dose of lawsone (Fig. 1c). At 25 µg/mL, violacein production was inhibited by 26.33% ± 4.58%. The inhibition increased to 50.72% ± 6.93% at 50 µg/mL and further to 79.74% ± 3.62% at 100 µg/mL. The strongest suppression was recorded at 250 µg/mL, where violacein production decreased by 90.62% ± 4.02%. These findings demonstrate that lawsone interferes with QS-regulated violacein biosynthesis in *C. violaceum*. These findings align with previous reports on structurally related naphthoquinones such as plumbagin (5-hydroxy-2-methyl-1,4-naphthoquinone), which inhibited violacein production by more than 80% at 10 µg/mL, with no significant impact on growth (26). While plumbagin exhibited better activity at lower doses, lawsone demonstrates comparable efficacy at higher sub-MIC levels.

The violacein inhibition was also performed in *C. violaceum* CV026 to check whether lawsone directly interferes with QS receptor activity, since CV026 cannot make its own AHLs and only produces violacein when supplied externally. The CV026 did not produce violacein in the absence of C_6_-HSL, but the pigment production was found in wells containing C_6_-HSL. At 25 µg/mL lawsone, violacein inhibition was low (15.50% ± 2.66%) but increased with higher doses: 23.35% ± 2.73% at 50 µg/mL, 41.26% ± 4.53% at 100 µg/mL, and reached 71.05% ± 5.75% at 250 µg/mL (Fig. 1c). These results demonstrate that lawsone interferes with QS signaling in CV026 even when AHLs are supplied externally. Since CV026 cannot synthesize its own AHLs, the observed inhibition shows that lawsone acts at the receptor level, likely by competing with or blocking CviR binding to AHLs, thereby preventing activation of violacein biosynthesis. However, the inhibition in *C. violaceum* CV026 (71.05% ± 5.75%) was lower compared to *C. violaceum* 12472 (90.62% ± 4.02%). These assays provide mechanistic evidence that lawsone functions as a QS inhibitor by targeting both LuxR-type receptors and reducing AHL synthesis.

### Lawsone inhibits QS-controlled virulence factors in *P. aeruginosa*

Pyocyanin, a phenazine-derived pigment, is a known virulence factor of *P. aeruginosa* (27). In untreated cultures, pyocyanin levels remained high (7.12 ± 0.44 µg/mL), whereas treatment with lawsone resulted in a clear concentration-dependent suppression as shown in Fig. 1d. At 50, 100, 250, and 500 µg/mL, pyocyanin inhibition was 29.82% ± 5.03%, 45.03% ± 4.83%, 78.32% ± 4.16%, and 87.90% ± 3.66%, respectively. Similar findings have been reported for plumbagin (another naphthoquinone), which inhibited pyocyanin biosynthesis by >60% at 125 µg/mL (26). Pyocyanin is known to impair host immune responses by inducing oxidative stress, apoptosis in neutrophils, and disruption of ciliary function (28–30). It also plays a key role in biofilm establishment (31). Therefore, the inhibition observed suggests that certain naphthoquinones interfere with QS-regulated phenazine biosynthesis, potentially attenuating multiple pathogenic traits simultaneously.

Pyoverdin, a fluorescent siderophore produced by *P. aeruginosa*, is known for iron acquisition and causing virulence (32). Lawsone treatment led to inhibition of pyoverdin secretion, with reductions of 23.77% ± 4.13%, 37.40% ± 3.13%, 64.57% ± 5.86%, and 81.97% ± 3.25% at 50, 100, 250, and 500 µg/mL, respectively (Fig. 1e). Pyoverdin binds host transferrin, depriving tissues of iron and facilitating colonization (33). Its suppression by lawsone indicates disruption of iron-scavenging mechanisms, which are critical for bacterial survival in host environments. This effect may weaken *P. aeruginosa* pathogenicity, particularly in iron-limited niches such as cystic fibrosis lungs.

Proteolytic enzymes, including LasA protease, are QS-regulated and contribute to tissue invasion and immune evasion (34, 35). Lawsone markedly reduced protease activity in a dose-dependent manner, with inhibition levels of 44.54% ± 3.06%, 55.15% ± 2.01%, 73.96% ± 5.11%, and 90.57%± 4.10% at 50, 100, 250, and 500 µg/mL, respectively (Fig. 1f). Since proteases degrade host proteins and facilitate dissemination, their suppression strongly suggests that lawsone interferes with lasI-lasR signaling. This disruption could significantly reduce the bacterium’s ability to damage host tissues and evade immune clearance. Elastase LasB, another QS-regulated enzyme, was also inhibited by lawsone. Reductions of 29.19% ± 6.12%, 42.14% ± 5.97%, 61.31% ± 3.40%, and 75.76% ± 4.17% were observed at 50, 100, 250, and 500 µg/mL, respectively (Fig. 1g). Elastases degrade extracellular matrix components and impair host immune functions (36). Their inhibition highlights the potential of lawsone to attenuate tissue damage caused by such enzymes. Given that elastase expression is tightly linked to QS circuits, these findings validate the possible role of lawsone as a QS disruptor.

The findings of protease inhibition in *P. aeruginosa* PAO1 are consistent with earlier reports on structurally related naphthoquinones. For instance, it was demonstrated that plumbagin significantly reduced azocasein-degrading protease activity in *P. aeruginosa* MTCC 2488 and *P. aeruginosa* MTCC 424, with nearly 60% and 40% inhibition, respectively (27). Moreover, plumbagin has also been found to inhibit the protease and elastase activity in *P. aeruginosa* PAO1 (26). These studies highlight that structurally related naphthoquinones, including lawsone and plumbagin, attenuate QS-regulated hydrolytic enzymes at sub-MIC levels.

Rhamnolipids, amphiphilic biosurfactants, are crucial for motility, biofilm formation, and surface colonization (37, 38). Lawsone suppressed rhamnolipid production by 19.73% ± 4.28%, 29.71% ± 5.11%, 43.68% ± 3.67%, and 52.21% ± 4.48% at 50, 100, 250, and 500 µg/mL, respectively (Fig. 1h). Since rhamnolipids facilitate swarming motility and biofilm structural integrity, their reduction indicates that lawsone impairs cooperative behaviors essential for infection establishment. The observed inhibition of rhamnolipid production in *P. aeruginosa* PAO1 is consistent with interference in the Rhl QS circuit, since rhamnolipid biosynthesis is primarily regulated by RhlR (39). This phenotypic suppression aligns with our qRT-PCR data, which showed significant downregulation of both *rhlI* and *rhlR* transcripts. These findings suggest that lawsone not only attenuates the Las system but also impacts the Rhl regulatory system. This effect of lawsone on both Las and Rhl circuits provides an explanation for the broad reduction in QS-controlled virulence traits.

The qRT-PCR showed that treatment with 500 µg/mL lawsone caused clear downregulation of key QS genes in *P. aeruginosa* PAO1. Relative to the control, the measured fold changes in *lasI*, *lasR*, *rhlI*, and *rhlR* were 0.423 ± 0.024, 0.243 ± 0.044, 0.547 ± 0.026, and 0.210 ± 0.031, respectively (Fig. 1i). The transcription of all four genes was reduced, with *lasR* and *rhlR* showing the strongest suppression (~75%–80% decrease), while *lasI* and *rhlI* showed moderate decreases (~45%–55%). These results indicate that lawsone acts on both the Las and Rhl QS circuits. The larger reduction in the receptor genes (*lasR* and *rhlR*) suggests that lawsone may more strongly affect transcriptional regulators or their stability, which would, in turn, reduce downstream effector synthesis. This transcriptional suppression is consistent with the decreased QS-regulated phenotypic data.

This study is focused on the anti-QS and antibiofilm potential of lawsone. However, the redox-cycling activity and generation of ROS by naphthoquinones, including lawsone, cannot be ignored, considering the compound for human applications. For example, lawsone has been reported to activate the SoxRS regulon in *Escherichia coli* at 0.2 mM (40). At higher concentrations, such activity raises concerns about oxidative stress and potential cytotoxicity. The nanoemulsified formulations of lawsone displayed cytotoxicity in human cell lines (3T3/NIH fibroblast cells), though with improved safety profiles compared to the free compound (41). These findings highlight both the therapeutic potential and the need for careful toxicity evaluation.

### Lawsone inhibits QS-controlled virulence factors in *S. marcescens*

**Fig 2:**
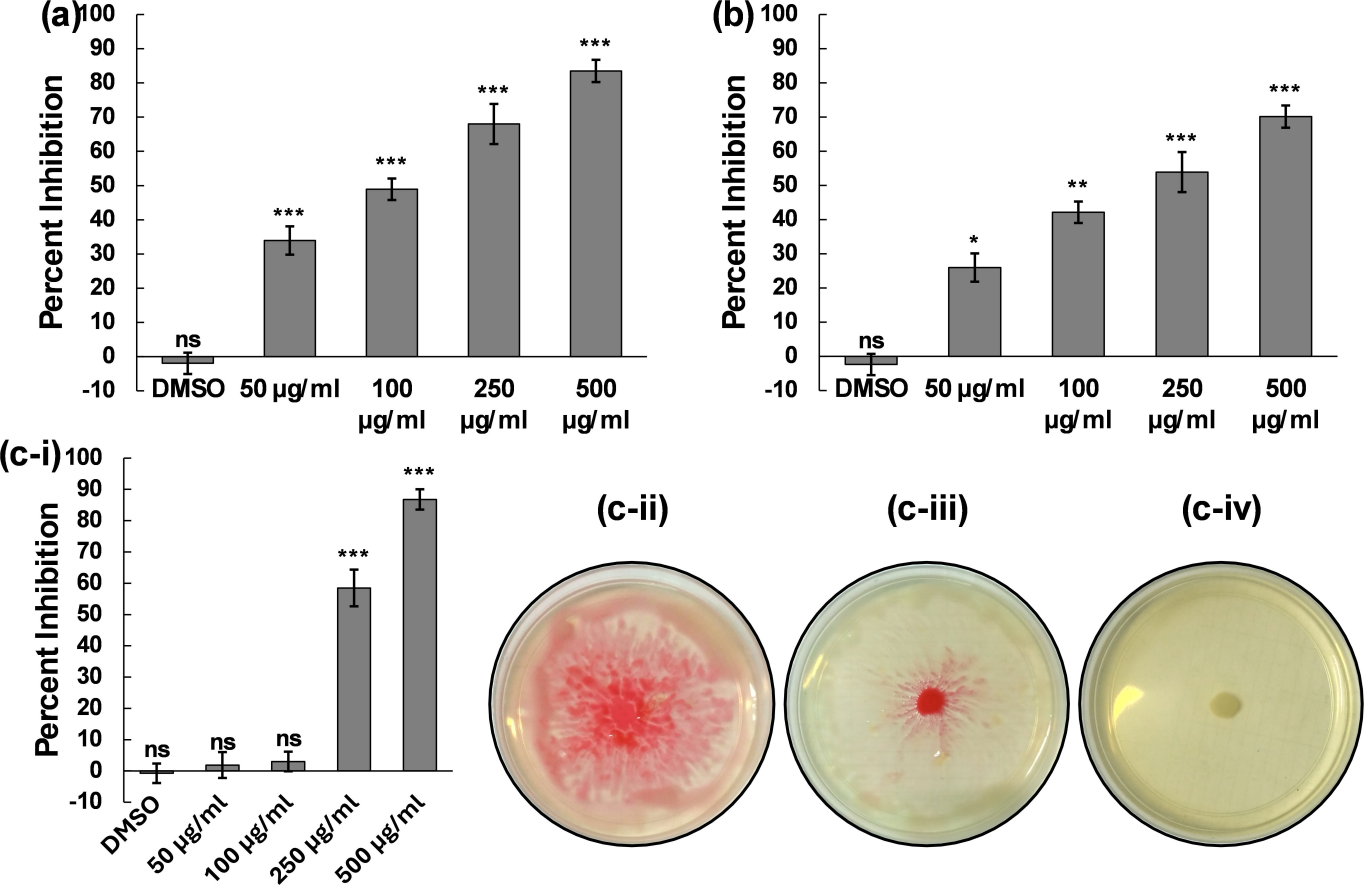
(**a**) Inhibition of prodigiosin in *S. marcescens* by lawsone. (**b**) Inhibition of protease in *S. marcescens* by lawsone. (**c**-i) Inhibition of swarming in *S. marcescens* by lawsone. (**c**-ii) Swarming of control *S. marcescens*. (**c**-iii) Swarming of *S. marcescens* treated with 250 µg/mL lawsone. (**c**-iv) Swarming of *S. marcescens* treated with 500 µg/mL lawsone. Results are expressed as mean ± SD. Statistical significance was determined using Student’s *t*-test. *P-value < 0.05; **P-value < 0.01; ***P-value < 0.001; and ns, statistically insignificant (*P*-value > 0.05). 50 µg/mL, 0.287 mM; 100 µg/mL, 0.574 mM; 250 µg/mL, 1.435 mM; and 500 µg/mL, 2.87 mM.

Prodigiosin is a characteristic red pigment produced by *S. marcescens*, and its biosynthesis is regulated by QS (42). In untreated cultures, pigment production was robust, whereas treatment with lawsone resulted in pigment suppression (Fig. 2a). At 50 µg/mL, prodigiosin was reduced by 33.94% ± 4.45%. The inhibition increased to 48.93% ± 5.16% at 100 µg/mL, 67.99% ± 6.10% at 250 µg/mL, and reached a maximum of 83.50% ± 3.23% at 500 µg/mL. Previous studies have suggested that prodigiosin biosynthesis is not an isolated trait but is interconnected with other QS-regulated functions, including protease secretion, flagellar morphology, and hemagglutination (43). The strong inhibition observed here indicates that lawsone interferes with AHL-mediated signaling, thereby suppressing pigment biosynthesis. Since prodigiosin has been implicated in bacterial survival, stress tolerance, and virulence, its reduction highlights the potential of lawsone to attenuate pathogenicity at multiple levels.

Lawsone treatment significantly reduced protease secretion (Fig. 2b). At 50 µg/mL, protease activity was inhibited by 25.97% ± 4.13%. The inhibition increased to 42.14% ± 3.13% at 100 µg/mL, 53.90% ± 5.86% at 250 µg/mL, and reached 70.13% ± 3.25% at 500 µg/mL. Proteases secreted by *S. marcescens* are known to degrade host proteins, modulate immune responses, and facilitate tissue invasion (44). Their production is regulated by multiple AHLs, including 3-oxo-C_6_-HSL, C_6_-HSL, C_7_-HSL, and C_8_-HSL, which also control prodigiosin synthesis and motility (45). The observed inhibition of protease activity by lawsone suggests that it disrupts QS signaling. The inhibition is important as proteases are considered crucial determinants of pathogenicity in hospital-acquired infections caused by *S. marcescens*.

Swarming motility, a flagella-driven surface movement, is a virulence trait of *S. marcescens* and plays a critical role in colonization of medical devices such as urinary catheters (46). On control plates without lawsone, *S. marcescens* swarmed freely, covering large areas of the agar surface (Fig. 2c). At 50 and 100 µg/mL, motility was not inhibited, and *S. marcescens* swarmed to the entire plate. However, the development of red pigment was reduced at these concentrations. At higher doses of 250 and 500 µg/mL, the inhibition was found to be 58.49% ± 4.57% and 86.79% ± 2.35%, respectively. Swarming motility is associated with biofilm formation, as it facilitates initial surface attachment and colonization (47). The strong suppression of swarming by lawsone, therefore, indicates that it may impair biofilm development. Since motility, pigment production, and protease secretion are all QS-regulated, the simultaneous inhibition of these traits strongly supports that lawsone may function as a putative competitive QS inhibitor.

These findings demonstrate that lawsone exerts broad-spectrum inhibition of QS-regulated virulence traits in *S. marcescens*. The dose-dependent suppression of prodigiosin, protease activity, and swarming motility highlights its ability to interfere with AHL-mediated signaling pathways. Importantly, these inhibitory effects were observed at sub-MIC levels, suggesting that lawsone attenuates pathogenicity without exerting direct bactericidal pressure. Moreover, the results align with earlier reports of plant-derived compounds acting as QS inhibitors in gram-negative pathogens (48).

### Lawsone inhibits biofilm formation

**Fig 3:**
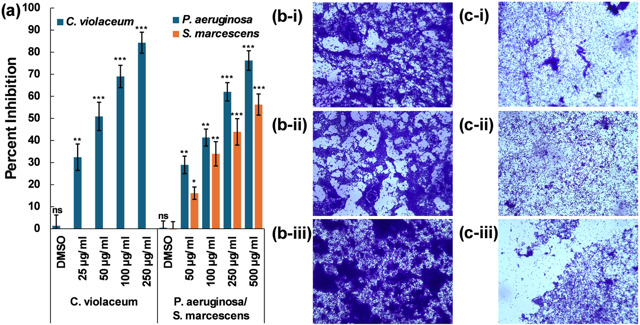
(**a**) Inhibition of biofilm formation in *C. violaceum*, *P. aeruginosa*, and *S. marcescens* by lawsone. (**b**-i) Control *C. violaceum* biofilm. (**b**-ii) Control *P. aeruginosa* biofilm. (**b**-iii) Control *S. marcescens* biofilm. (**c**-i) 250 µg/mL lawsone-treated *C. violaceum* biofilm. (**c**-ii) 500 µg/mL lawsone-treated *P. aeruginosa* biofilm. (**c**-iii) 500 µg/mL lawsone-treated *S. marcescens* biofilm. Results are expressed as mean ± SD. Statistical significance was determined using Student’s *t*-test. *P-value < 0.05; **P-value < 0.01; ***P-value < 0.001; and ns, statistically insignificant (*P*-value > 0.05). 25 µg/mL, 0.143 mM; 50 µg/mL, 0.287 mM; 100 µg/mL, 0.574 mM; 250 µg/mL, 1.435 mM; and 500 µg/mL, 2.87 mM.

The treatment with lawsone caused a reduction in biofilm development in the tested bacteria (Fig. 3a). In *C. violaceum*, biofilm was reduced by 32.43% ± 5.98%, 50.89% ± 6.41%, 68.98% ± 5.07%, and 84.28% ± 4.76% at 25, 50, 100, and 250 µg/mL, respectively. A similar suppressive trend was observed in *P. aeruginosa*, where biofilm inhibition reached 28.94% ± 3.96%, 41.32% ± 3.85%, 62.03% ± 4.15%, and 76.22% ± 4.41% at 50, 100, 250, and 500 µg/mL, respectively. In *S. marcescens*, the reductions were comparatively lower but still significant, with inhibition levels of 16.12% ± 2.80%, 33.96% ± 5.50%, 43.89% ± 5.97%, and 56.27% ± 4.77% at 50, 100, 250, and 500 µg/mL, respectively. The findings are consistent with earlier reports on naphthoquinone derivatives as biofilm inhibitors. For example, 2-hydroxy-3-substituted naphthoquinones (compounds 9e, 9f, 9j, and 9k) were shown to inhibit *P. aeruginosa* ATCC 15442 biofilm formation at sub-MICs (1/8× to 1/2× MIC) without affecting bacterial growth. Notably, derivative 9j reduced biofilm biomass by nearly 65% at 1/2× MIC (49).

Microscopic observations further validated these quantitative findings. Untreated cultures of all three pathogens formed dense biofilms on glass coverslips, characterized by compact clusters and strong adherence (Fig. 3b). In contrast, lawsone-treated cultures displayed a scattered appearance, with fewer adherent cells and loss of the characteristic cluster-like structures (Fig. 3c). Most of the treated cells remained planktonic, indicating that lawsone interferes with microbial adherence and subsequent biofilm maturation.

Biofilm formation is a virulence trait, protecting bacteria against antibiotics, host immune responses, and environmental stresses (50, 51). The structural integrity of biofilms is largely dependent on extracellular polymeric substances (EPS), which act as a scaffold for the microbial communities. Since EPS production and biofilm development are regulated by QS signaling (52), the observed inhibition strongly suggests that lawsone disrupts AHL-mediated QS. Biofilms are implicated in nearly 80% of human infections, including chronic respiratory diseases, urinary tract infections, and device-associated infections. In *P. aeruginosa*, biofilm formation is particularly problematic in cystic fibrosis patients, where it contributes to persistent colonization and antibiotic resistance. Similarly, *S. marcescens* biofilms are associated with catheter-related urinary tract infections, while *C. violaceum* is a model organism for QS studies. By attenuating biofilm formation at sub-MICs, lawsone demonstrates its potential as an antibiofilm agent. However, further studies in antibiotic-resistant clinical isolates and in animal models are needed to establish its therapeutic applicability.

### Molecular docking

Molecular docking was performed to evaluate the binding affinity and interaction profile of lawsone with QS regulators SmaR (*S. marcescens*), LasR (*P. aeruginosa*), and CviR (*C. violaceum*). First, the docking method was validated by extracting the native ligand C_6_-HSL from CviR and then redocking. The ligand was found to be docked in the same binding site as it was present in the crystal structure, validating the docking methodology. The docking results revealed that lawsone fits into the ligand-binding pockets of all three proteins, with binding energies ranging between −7.7 and −8.5 kcal/mol (Table 1). These values indicate a stable interaction, comparable to other reported natural QS inhibitors.

**TABLE 1:** Binding energies and details for the interaction of lawsone with CviR, LasR, and SmaR obtained using molecular docking

Complex	Binding energy (kcal/mol)	Hydrogen bond	Van der Waals forces	Hydrophobic forces	Ionic forces
CviR-lawsone complex	−7.8	Ser155, Trp84, Tyr88, and Tyr80	Leu57, Leu85, Met135, Phe126, Phe115, and Thr140	Ile99, Ala130, and Trp111	Asp97
LasR-lawsone complex	−8.5	Ser129, Thr75, Thr115, Trp60, and Tyr56	Trp88, Phe101, Arg61, and Ala127	Ala105, Leu110, Leu36, and Tyr64	Asp73
SmaR-lawsone complex	−7.7		Ala72, Phe44, Ile46, Phe54, Trp53, Met126, Trp81, Ser124, and Thr110	Ala34, Tyr57, Ala32, Val122, Leu69, and Val68	Asp66

**Fig 4:**
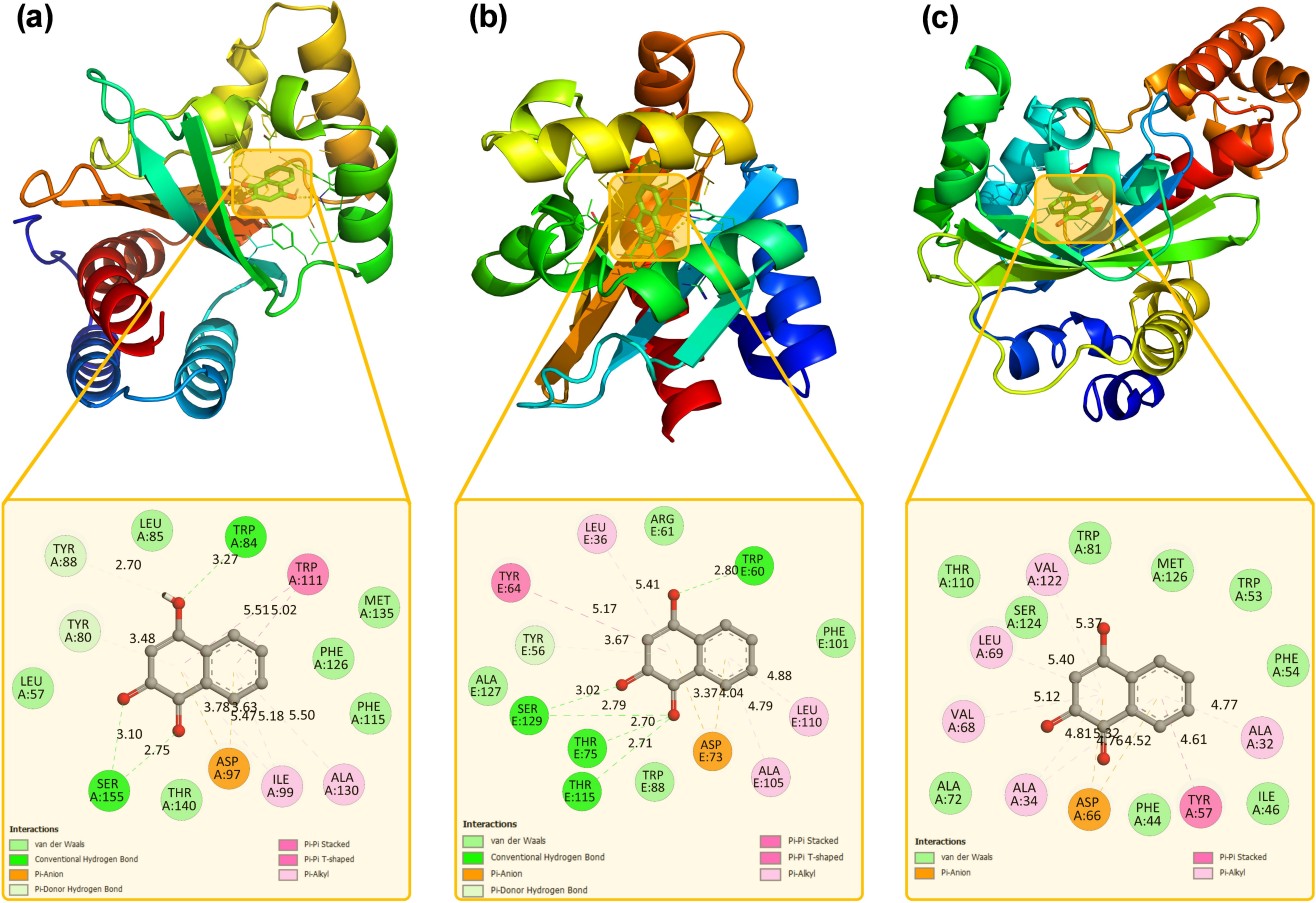
(**a**) Molecular docked complex of lawsone with CviR. (**b**) Molecular docked complex of lawsone with LasR. (**c**) Molecular docked complex of lawsone with SmaR. The conformation with the lowest energy was selected for analysis and representation. The molecular docking was performed with AutoDock Vina.

CviR is a LuxR-type transcriptional regulator that recognizes C_6_-HSL autoinducers to activate violacein biosynthesis in *C. violaceum* (53). Lawsone exhibited a binding energy of −7.8 kcal/mol, which was slightly higher than the redocking of native ligand C_6_-HSL to CviR (−7.4 kcal/mol), suggesting that lawsone has a slightly higher affinity than the native ligand. Hydrogen bonds were formed with Ser155, Trp84, Tyr88, and Tyr80 (Fig. 4a), the residues known to stabilize the native ligand. Van der Waals contacts were observed with Leu57, Leu85, Met135, Phe126, Phe115, and Thr140, while hydrophobic interactions involved Ile99, Ala130, and Trp111. An ionic bond was established with Asp97, a residue previously reported to anchor the acyl tail of native AHLs (53). The docking results suggest that lawsone may be a possible competitive inhibitor, occupying the autoinducer-binding domain and preventing native AHLs from triggering violacein synthesis (54).

LasR is the key QS regulator in *P. aeruginosa*, activated by 3-oxo-C_12_-HSL, and governs the expression of multiple virulence factors, including pyocyanin, elastase, and rhamnolipids (55). Lawsone displayed the strongest binding affinity among the tested proteins, with a binding energy of −8.5 kcal/mol. The binding energy for the redocking of 3-oxo-C_12_-AHL LasR was −8.8 kcal/mol. Hydrogen bonds were formed with Ser129, Thr75, Thr115, Trp60, and Tyr56, indicating strong polar interactions within the ligand-binding pocket (Fig. 4b). Van der Waals forces were observed with Trp88, Phe101, Arg61, and Ala127, while hydrophobic contacts involved Ala105, Leu110, Leu36, and Tyr64. An ionic interaction with Asp73 further stabilized the complex. The combination of hydrogen bonding, hydrophobic packing, and electrostatic interactions suggests that lawsone can effectively compete with native AHLs, thereby interfering with LasR-mediated transcriptional activation. Since LasR is also involved in biofilm formation required for the establishment of infection, lawsone’s binding to LasR may prevent the QS-dependent virulent traits.

SmaR is a LuxR-type regulator in *S. marcescens*, involved in controlling prodigiosin biosynthesis, protease secretion, and motility. Lawsone exhibited a binding energy of −7.7 kcal/mol, indicating moderate affinity. Numerous van der Waals interactions were observed with Ala72, Phe44, Ile46, Phe54, Trp53, Met126, Trp81, Ser124, and Thr110 (Fig. 4c). Hydrophobic contacts were formed with Ala34, Tyr57, Ala32, Val122, Leu69, and Val68, while an ionic interaction was noted with Asp66. The absence of hydrogen bonding suggests that lawsone stabilizes the complex primarily through hydrophobic and van der Waals forces. This mode of binding might block native AHLs, thereby attenuating QS-regulated virulence traits in *S. marcescens*.

The docking results demonstrate that lawsone interacts with QS regulators of three gram-negative pathogens through a combination of hydrophobic contacts, hydrogen bonding, van der Waals contacts, and ionic forces. These findings suggest that lawsone may be a possible competitive inhibitor that occupies ligand-binding domains of LuxR-type proteins and hence prevents the native autoinducers from activating QS pathways. Given that QS controls a wide range of virulence traits, the ability of lawsone to disrupt these protein-ligand interactions highlights its potential as a broad-spectrum anti-QS agent. By targeting regulatory proteins rather than bacterial viability, lawsone offers a promising strategy to attenuate pathogenicity in gram-negative bacteria.

### Molecular dynamics simulations

**Fig 5:**
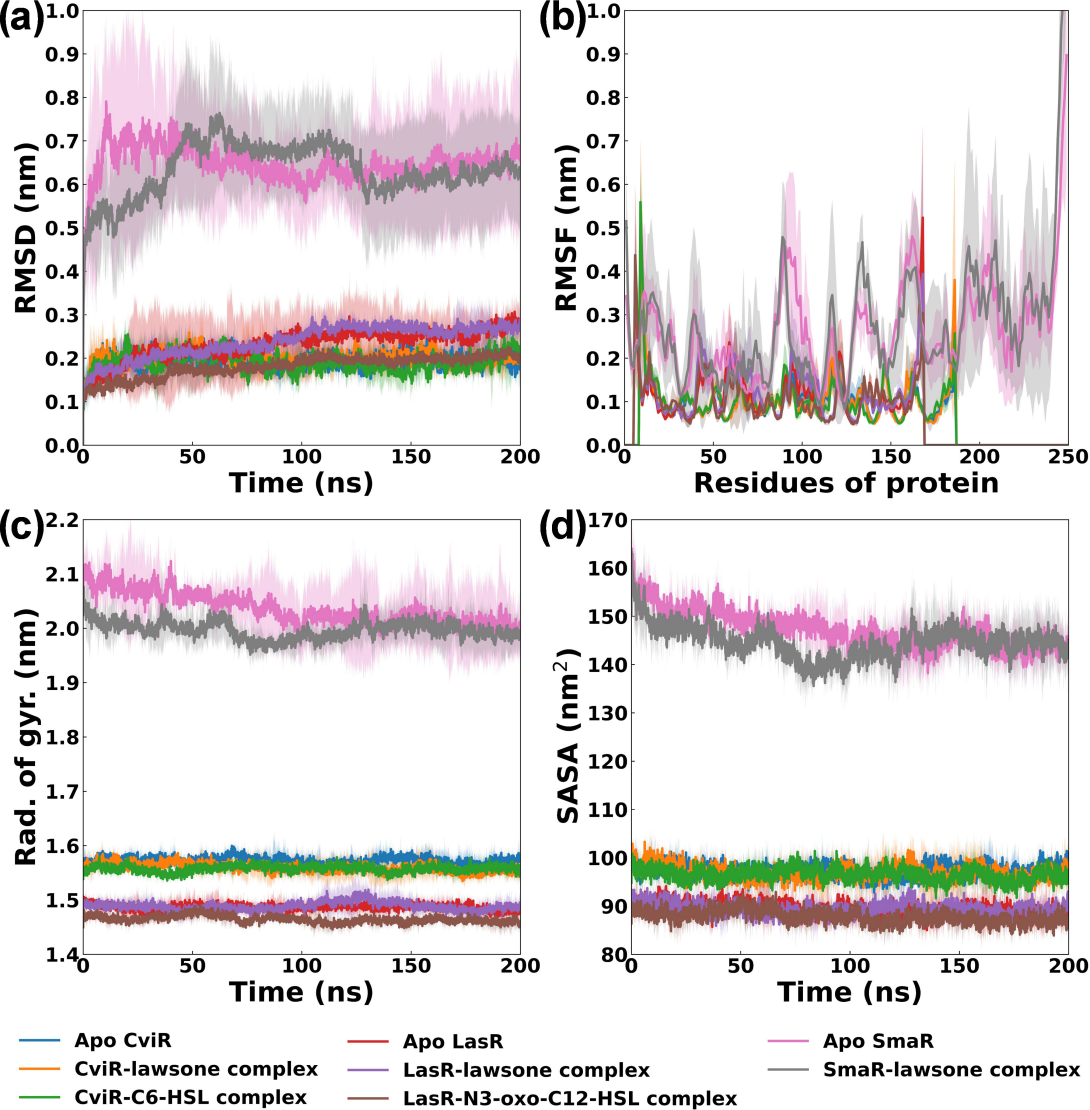
(**a**) RMSD of backbone atoms of apo CviR, CviR-lawsone complex, CviR-C_6_-HSL complex, apo LasR, LasR-lawsone complex, LasR-N3-oxo-C_12_-HSL complex, apo SmaR, and SmaR-lawsone complex over 100 ns MD simulation. (**b**) RMSF of alpha carbon atoms CviR, LasR, and SmaR without or with lawsone. (**c**) Radius of gyration of backbone atoms of apo CviR, CviR-lawsone complex, CviR-C_6_-HSL complex, apo LasR, LasR-lawsone complex, LasR-N3-oxo-C_12_-HSL complex, apo SmaR, and SmaR-lawsone complex over 100 ns MD simulation. (**d**) SASA of apo CviR, CviR-lawsone complex, CviR-C_6_-HSL complex, apo LasR, LasR-lawsone complex, LasR-N3-oxo-C_12_-HSL complex, apo SmaR, and SmaR-lawsone complex over 100 ns MD simulation. The data are presented as the average of three MD simulations with standard deviations.

Following molecular docking, MD simulations were conducted to evaluate the dynamic behavior and stability of apo proteins and their ligand-bound complexes. Root mean square deviation (RMSD) trajectories serve as a measure of structural deviation during simulation. As shown in Fig. 5a, all systems attained equilibrium within the first half of the simulation duration, indicating stable trajectories. The average RMSD for apo CviR was 0.194 ± 0.016 nm, which remained nearly unchanged upon binding with lawsone (0.200 ± 0.016 nm) or native C_6_-HSL (0.189 ± 0.020 nm). Similarly, LasR exhibited RMSD values of 0.231 ± 0.034 nm in apo form, 0.238 ± 0.036 nm when bound to lawsone, and 0.181 ± 0.023 nm with native 3-oxo-C_12_-HSL. SmaR displayed higher RMSD values compared to CviR and LasR, with 0.648 ± 0.041 nm in apo form and 0.632 ± 0.061 nm in lawsone-bound complex, reflecting its larger and more flexible structure. These results suggest that lawsone binding did not induce major structural changes, and proteins retained their conformational stability throughout the simulation. Residue-level flexibility was assessed using RMSF values of C_α_ atoms (Fig. 5b). For CviR, average RMSF values were 0.108 ± 0.052 nm in apo form, 0.107 ± 0.054 nm in lawsone-bound complex, and 0.107 ± 0.054 nm in C_6_-HSL complex, indicating negligible differences. LasR showed slightly higher fluctuations, with RMSF values of 0.119 ± 0.065 nm (apo), 0.126 ± 0.061 nm (lawsone-bound), and 0.110 ± 0.051 nm (native ligand-bound). These fluctuations were primarily observed in loop regions, which are inherently dynamic in aqueous environments. SmaR exhibited relatively higher RMSF values, with 0.112 ± 0.261 nm in apo form and 0.147 ± 0.272 nm in lawsone-bound complex, reflecting its flexible architecture. Overall, the RMSF profiles indicate that lawsone binding did not markedly alter backbone mobility, and the proteins remained stable.

*R*_*g*_ values provide a measure of protein compactness during simulation. The apo CviR recorded an average *R*_*g*_ of 1.572 ± 0.006 nm, which slightly decreased to 1.559 ± 0.007 nm upon lawsone binding and 1.557 ± 0.006 nm with C_6_-HSL (Fig. 5c). Apo LasR exhibited an *R*_*g*_ of 1.486 ± 0.005 nm, which remained stable at 1.488 ± 0.007 nm with lawsone and 1.464 ± 0.006 nm with native ligand. SmaR showed higher *R*_*g*_ values due to its larger size, with 2.036 ± 0.028 nm in apo form and 1.996 ± 0.015 nm in lawsone-bound complex. These minor shifts suggest that lawsone binding did not compromise protein compactness, and the overall structural integrity was maintained. SASA values reflect the level of surface exposure, which can indicate conformational changes upon ligand binding. Apo CviR exhibited SASA of 97.36 ± 1.33 nm^2^, which slightly decreased to 96.95 ± 1.41 nm^2^ with lawsone and 96.57 ± 1.29 nm^2^ with C_6_-HSL (Fig. 5d). Apo LasR recorded SASA of 89.49 ± 1.25 nm^2^, which remained nearly unchanged at 89.37 ± 1.22 nm^2^ with lawsone and slightly reduced to 87.82 ± 1.34 nm^2^ with native ligand. SmaR displayed higher SASA values, consistent with its larger structure, with 147.25 ± 3.78 nm^2^ in apo form and 144.69 ± 3.34 nm^2^ in lawsone-bound complex. These subtle changes indicate that lawsone binding did not significantly alter protein surface exposure.

**Fig 6:**
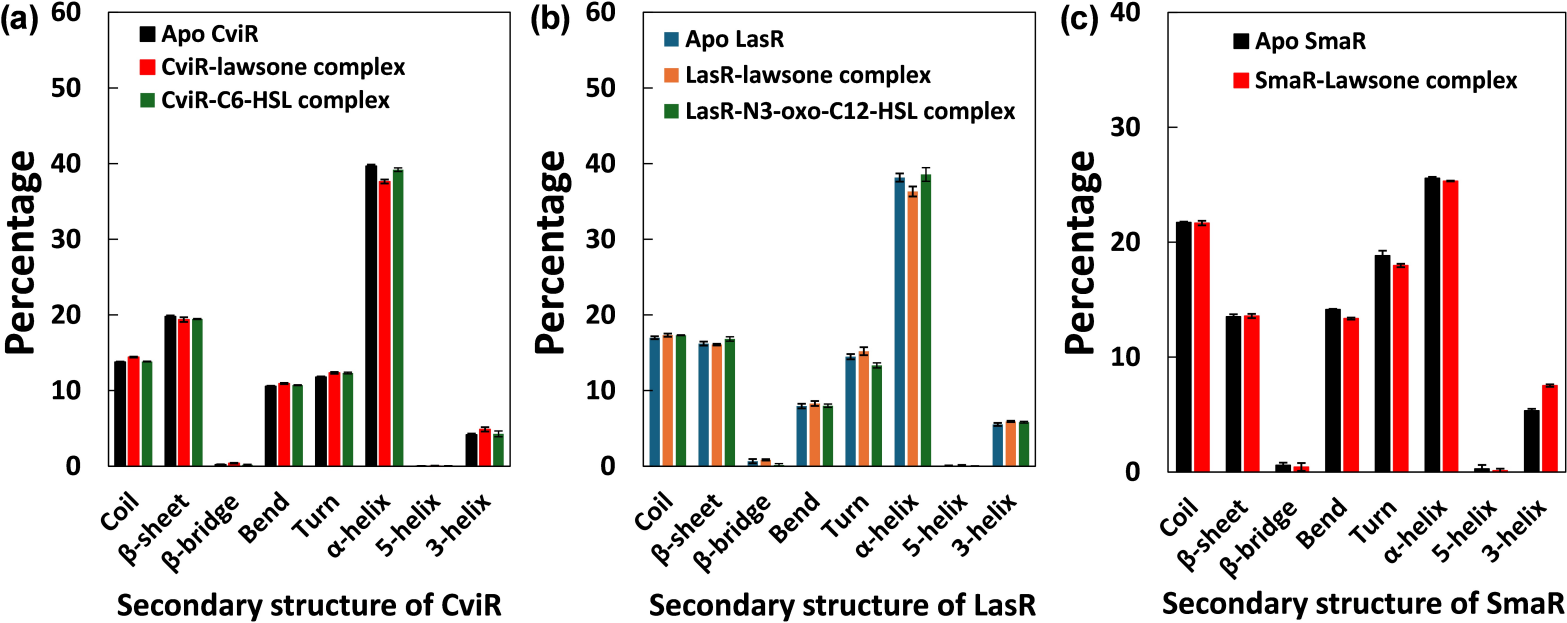
(**a**) Secondary structural components in CviR without and with lawsone or CviR-C_6_-HSL. (**b**) Secondary structural components in LasR without and with lawsone or LasR-N3-oxo-C_12_-HSL. (**c**) Secondary structural components in SmaR without and with lawsone. The data are presented as the average of three MD simulations with standard deviations.

To evaluate the influence of lawsone on the structural integrity of QS regulators, the secondary structure composition of apo proteins and their ligand-bound complexes was analyzed (Fig. 6). The apo form of CviR exhibited 13.78% coils, 19.80% β-sheets, 0.21% β-bridges, 10.56% bends, 11.79% turns, and 39.68% α-helices (Fig. 6a). Upon binding with lawsone, these values shifted only marginally to 14.42% coils, 19.38% β-sheets, 0.40% β-bridges, 10.93% bends, 12.33% turns, and 37.63% α-helices. The native C_6_-HSL complex displayed similar proportions, with 13.84% coils, 19.46% β-sheets, and 39.19% α-helices. LasR in its apo form displayed 16.99% coils, 16.21% β-sheets, 0.67% β-bridges, 7.95% bends, 14.48% turns, and 38.16% α-helices (Fig. 6b). Lawsone binding resulted in slight increases in coil (17.32%) and turn (15.20%) content, while α-helices decreased modestly to 36.31%. The native 3-oxo-C_12_-HSL complex showed comparable values, with 17.31% coils, 16.82% β-sheets, and 38.56% α-helices. SmaR exhibited higher coil content compared to CviR and LasR, reflecting its inherently flexible nature. The apo protein (SmaR) displayed 21.73% coils, 13.51% β-sheets, 14.15% bends, 18.83% turns, and 25.57% α-helices (Fig. 6c). Lawsone binding produced only minor changes, with 21.67% coils, 13.58% β-sheets, 13.36% bends, 17.97% turns, and 25.33% α-helices. Interestingly, the three-helix content increased from 5.34% in apo form to 7.52% in the lawsone complex, suggesting subtle local rearrangements without compromising global folding. The observed variations in coil, bend, and turn elements were minor and within the expected dynamic range of proteins in aqueous environments. These findings strongly support that lawsone interacts stably with CviR, LasR, and SmaR, occupying their ligand-binding domains without disrupting native secondary structure.

**Fig 7:**
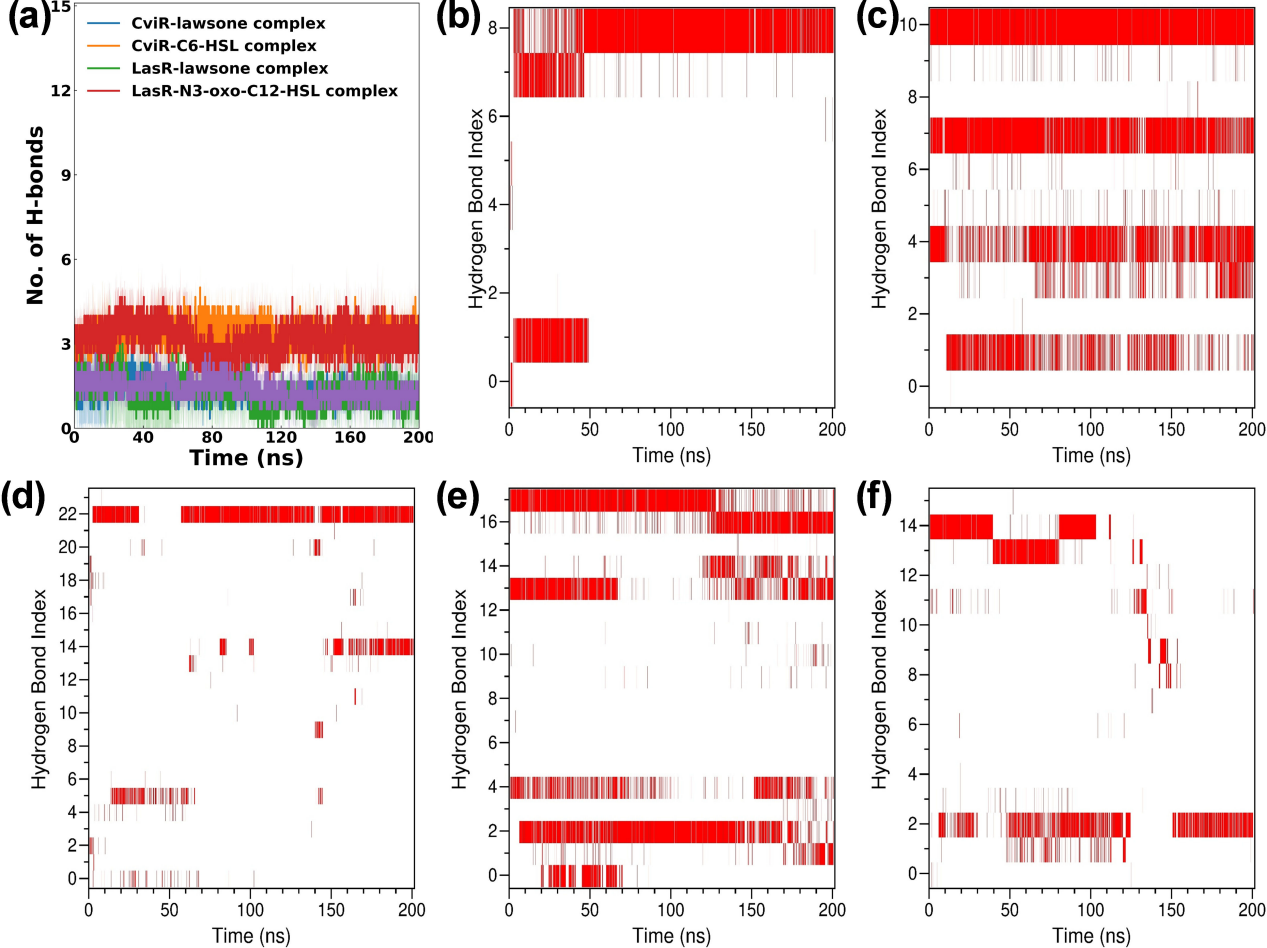
(**a**) Number of intermolecular hydrogen bonds between various complexes over 100 ns MD simulation. (**b**) Intermolecular hydrogen-bond existence map of CviR-lawsone complex. (**c**) Intermolecular hydrogen-bond existence map of CviR-C_6_-HSL complex. (**d**) Intermolecular hydrogen-bond existence map of LasR-lawsone complex. (**e**) Intermolecular hydrogen-bond existence map of LasR-N3-oxo-C_12_-HSL complex. (**f**) Intermolecular hydrogen-bond existence map of SmaR-lawsone complex. The data are presented as the average of three MD simulations with standard deviations.

To gain deeper insights into the molecular interactions of lawsone with QS regulators, hydrogen bond dynamics were analyzed during MD simulations (Fig. 7). Hydrogen bonds are critical stabilizing forces in protein-ligand complexes. Lawsone formed an average of 1.283 ± 0.350 hydrogen bonds with CviR, whereas the native C_6_-HSL ligand established a significantly higher average of 3.342 ± 0.384 hydrogen bonds. This difference suggests that while lawsone can occupy the binding pocket, its interaction relies more on hydrophobic and van der Waals contacts rather than extensive hydrogen bonding. Nevertheless, the presence of hydrogen bonds with residues such as Ser155, Trp84, and Asp97 supports the stability of the CviR-lawsone complex. LasR, the main QS regulator in *P. aeruginosa*, exhibited similar trends. Lawsone formed an average of 1.254 ± 0.459 hydrogen bonds, compared to 3.187 ± 0.516 hydrogen bonds formed by the native 3-oxo-C_12_-HSL ligand. The reduced hydrogen bonding indicates that lawsone stabilizes LasR primarily through hydrophobic and van der Waals interactions, consistent with docking results. Despite fewer hydrogen bonds, the overall binding energy of lawsone with LasR was favorable, suggesting that lawsone can act as a competitive inhibitor by occupying the ligand-binding domain without destabilizing the protein. In *S. marcescens*, lawsone formed an average of 1.370 ± 0.336 hydrogen bonds with SmaR. Given that SmaR regulates prodigiosin biosynthesis, protease secretion, and motility, the ability of lawsone to maintain consistent hydrogen bonding interactions highlights its potential to interfere with QS signaling in this pathogen.

**Fig 8:**
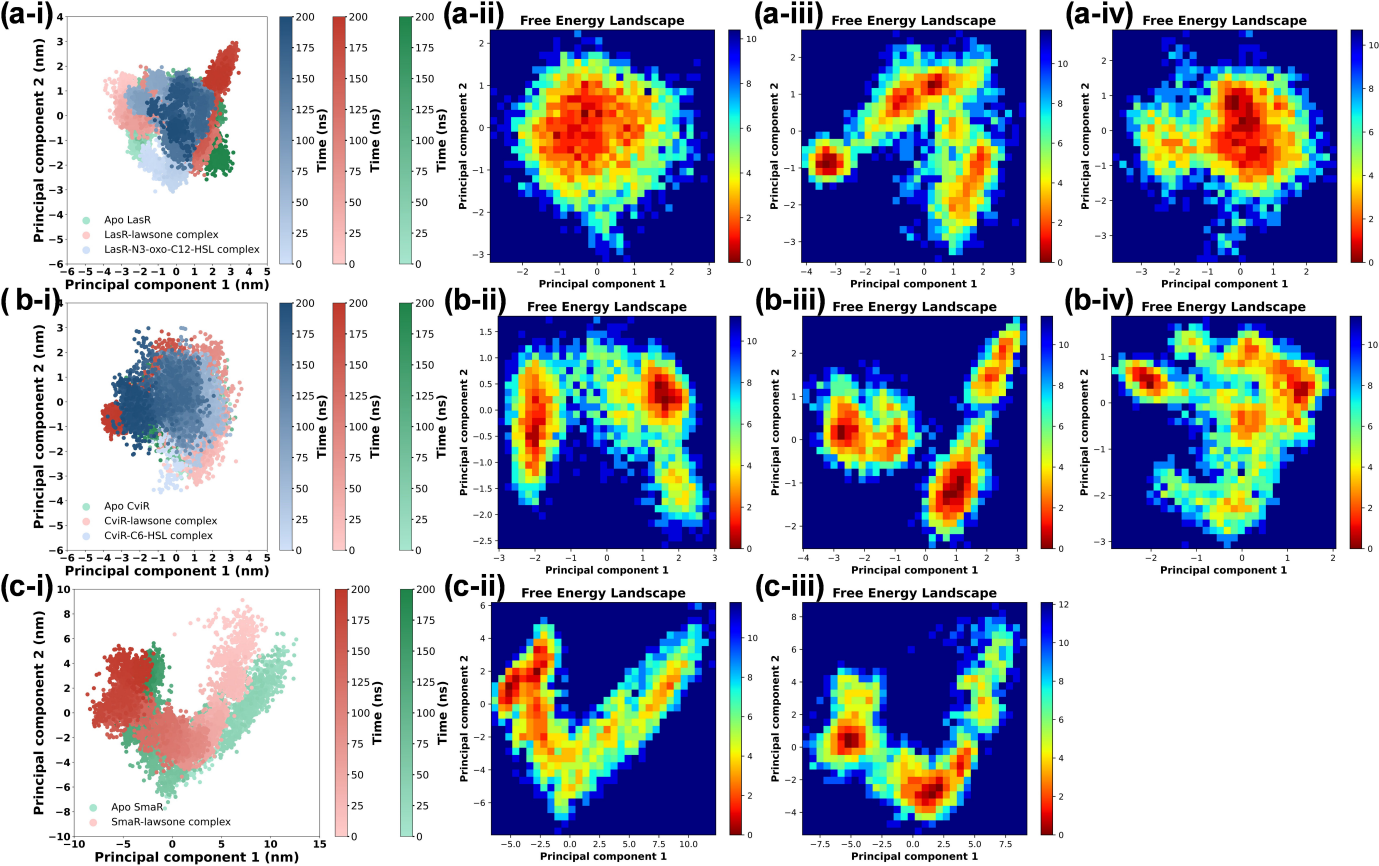
(**a**-i) Projection of eigenvectors of apo CviR, CviR-lawsone complex, and CviR-C_6_-HSL complex. (**a**-ii) Free energy landscape of apo CviR. (**a**-iii) Free energy landscape of CviR-lawsone complex. (**a**-iv) Free energy landscape of CviR-C_6_-HSL complex. (**b**-i) Projection of eigenvectors of apo LasR, LasR-lawsone complex, and LasR-N3-oxo-C_12_-HSL complex. (**b**-ii) Free energy landscape of apo LasR. (**b**-iii) Free energy landscape of LasR-lawsone complex. (**b**-iv) Free energy landscape of LasR-N3-oxo-C_12_-HSL complex. (**c**-i) Projection of eigenvectors of apo SmaR and SmaR-lawsone complex. (**c**-ii) Free energy landscape of apo SmaR. (**c**-iii) Free energy landscape of SmaR-lawsone complex.

Principal component analysis (PCA) was conducted to evaluate the flexibility of QS regulators in both their apo forms and ligand-bound states. Two principal components (eigenvectors) were computed, providing insights into the overall structural variations. The projection of these eigenvectors for the apo and ligand-bound forms is illustrated in Fig. 8. The spatial distribution of both complexes (CviR-lawsone and CviR-C_6_-HSL) exhibited a comparable range to that of apo CviR, suggesting that lawsone binding did not induce major deviations in global motions. Similarly, the 2D projection of eigenvectors for LasR-lawsone and LasR-3-oxo-C_12_-HSL complexes showed trajectories overlapping with apo LasR, indicating that the flexibility of LasR remained largely unchanged after ligand binding. Similarly, the eigenvector projections of lawsone complex were broadly similar to the apo SmaR, with subtle differences in distribution.

Following PCA, the eigenvectors were utilized to construct the free energy landscape (FEL), offering deeper insights into conformational stability. The FEL representations for all proteins and complexes are depicted in Fig. 8. Across all trajectories, energy minima were achieved, although their positions varied within the landscapes. Apo CviR displayed a single dominant energy minimum, while the CviR-lawsone complex exhibited two minima, and the native CviR-C_6_-HSL complex showed three minima. This suggests that lawsone binding introduced slightly higher conformational heterogeneity compared to the apo state, though still maintaining stable basins. For LasR, the apo protein exhibited two energy minima, whereas the LasR-lawsone complex displayed six distinct minima, indicating greater conformational flexibility upon lawsone binding. In contrast, the native LasR-3-oxo-C_12_-HSL complex showed only one minimum, reflecting a more stable conformational state. SmaR exhibited two energy minima in its apo form, consistent with its inherently flexible structure. Upon lawsone binding, the number of minima increased to four, suggesting enhanced conformational diversity. The PCA and FEL analyses revealed that lawsone binding did not significantly alter the global dynamics of CviR, LasR, or SmaR. The proteins retained their native flexibility profiles, and the energy landscapes confirmed stable conformations across all complexes.

To explore the thermodynamic aspects of lawsone binding with QS regulators, MM-PBSA computations were performed using representative frames from the MD trajectories. The binding free energy was decomposed into electrostatic, van der Waals, polar solvation, and SASA energies (Table 2). The CviR-lawsone complex exhibited an overall binding energy of −12.8 ± 0.80 kcal/mol, which was weaker compared to the native C_6_-HSL complex (−62.88 ± 8.50 kcal/mol). Van der Waals interactions contributed significantly (−19.69 ± 0.39 kcal/mol), while electrostatic forces were relatively minor (−4.68 ± 1.15 kcal/mol). LasR displayed stronger affinity for lawsone, with a binding energy of −14.59 ± 0.92 kcal/mol. In comparison, the native 3-oxo-C_12_-HSL complex exhibited a more favorable binding energy of −25.24 ± 0.11 kcal/mol. Van der Waals interactions (−20.21 ± 0.72 kcal/mol) were the dominant stabilizing force, while electrostatic contributions were modest (−4.35 ± 0.81 kcal/mol). The SmaR-lawsone complex exhibited a binding energy of −11.92 ± 1.34 kcal/mol, reflecting moderate affinity. Van der Waals interactions (−21.73 ± 1.24 kcal/mol) were the primary stabilizing factors, while electrostatic contributions (−10.32 ± 5.30 kcal/mol) were higher compared to CviR and LasR. Polar solvation energy was positive for all complexes, indicating an unfavorable contribution. These findings suggest that lawsone stabilizes the QS regulatory proteins CviR primarily through van der Waals contacts, with fewer electrostatic and SASA energy contributions.

**TABLE 2:** MM-PBSA binding energies for the interaction of ligands with CviR, LasR, and SmaR

Energy	CviR-lawsone complex	CviR-C_6_-HSL complex	LasR-lawsone complex	LasR-N3-oxo-C_12_-HSL complex	SmaR-lawsone complex
Van der Waals energy	−19.69 ± 0.39	−42.47 ± 3.64	−20.21 ± 0.72	−45.73 ± 1.25	−21.73 ± 1.24
Electrostatic energy	−4.68 ± 1.15	−147.07 ± 12.56	−4.35 ± 0.81	−13.44 ± 2.86	−10.32 ± 5.30
Polar solvation energy	14.19 ± 0.53	131.65 ± 5.50	12.69 ± 0.60	38.51 ± 4.21	22.69 ± 4.31
SASA energy	−2.67 ± 0.06	−4.98 ± 0.13	−2.72 ± 0.03	−4.56 ± 0.01	−2.58 ± 0.08
Binding energy	−12.87 ± 0.80	−62.88 ± 8.50	−14.59 ± 0.92	−25.24 ± 0.11	−11.92 ± 1.34

### Conclusion

This study shows the promising anti-QS and antibiofilm activity of lawsone against gram-negative bacteria. Lawsone reduced pigment production (violacein, pyocyanin, and prodigiosin), protease activity, motility, and biofilm formation in a dose-dependent manner. Computational analyses confirmed that lawsone binds stably to QS regulators CviR, LasR, and SmaR, mainly through van der Waals and hydrophobic interactions. MD simulations data further supported the stability of these complexes without disturbing protein structure. Together, these findings suggest that lawsone can act as a competitive inhibitor of QS signaling, weakening bacterial virulence and persistence. However, it is acknowledged that lawsone possesses redox-cycling activity and has been reported to induce oxidative stress and cytotoxicity in bacterial and mammalian systems. This ability of lawsone to induce cytotoxicity cannot be ignored. Future studies are therefore needed in animal models and mammalian cell systems to evaluate cytotoxicity, oxidative stress induction, and drug interaction potential before its translational application.

## Data Availability

All data supporting the findings of this study are included within the manuscript.
